# Hospitalization costs of treating colorectal cancer in China: A retrospective analysis

**DOI:** 10.1097/MD.0000000000016718

**Published:** 2019-08-16

**Authors:** Gao-Le Yuan, Li-Zhong Liang, Zhong-Fang Zhang, Qi-Lian Liang, Zhen-Yi Huang, Hui-Jie Zhang, Shao-Ang Cheng, Xiao-Xia Peng

**Affiliations:** a Oncology Center; b Medical Insurance Office, Affiliated Hospital of Guangdong Medical University, Zhanjiang; c School of Public Finance and Public Administration, Jiangxi University of Finance and Economics, Nanchang; d Department of finance, Central Hospital of Guangdong Agriculture Reclamation, Zhanjiang, China.

**Keywords:** colorectal cancer, health insurance, hospitalization costs, length of hospital stay

## Abstract

**Background:**

The objective of this study was to explore the influence factors of hospitalization costs of treating colorectal cancer in China. And the study provides new estimates on hospitalization costs and length of hospital stay for patients with colorectal cancer in China.

**Methods:**

Data for inpatient hospitalization associated with colorectal cancer were obtained from a 3-tier hospital in Guangdong Province and were analyzed post hoc. We conducted descriptive statistical methods, Wilcoxon rank–sum tests (for 2 groups) and the Kruskal–Wallis test (for more than 2 groups) to analyze the hospitalization costs of treating colorectal cancer.

**Results:**

The analysis included 8021 patients (female: 40.54%; mean age; 61.80 ± 13.28 years; male: 59.46%; mean age: 61.80 ± 13.28 years). The overall mean length of hospital stay was 11.35 days. Over the 5 years, the mean length of hospital stay showed a small decrease from 12.22 days in 2012 to 10.69 days in 2016, while per-day costs showed a trend of increase between 2012 and 2015 (increase from < 1190.94 to < 1382.50). The mean length of hospital stay was statistically significant difference was found for sexes (*P* = .039) and insurance status (*P* < .001). The mean hospitalization costs were < 16,279.58. Mean hospitalization costs were different among the UEBMI, the URBMI and the Unspecified (< 17,114.58, < 15,555.05, and < 17,735.30, respectively; *P* < .001).

**Conclusion:**

The study showed that hospitalization costs increase were associated with a small decreasing length of hospital stay and increasing per-day hospitalization costs. Moreover, the proportion of the hospitalization costs reimbursed by insurances increased. For inpatients with UEBMI, it possibly lead to over treatment and the medical expense rise which result in medical resources waste and significant society costs. The rising hospitalization costs may lead to a remarkably increased financial burden in the future in China.

## 1 Introduction

Colorectal cancer is one of the most common malignancies, and its morbidity and mortality in developed countries are ranked second and third, respectively.^[1,2]^ In recent years, the incidence of colorectal cancer in China has also increased, ranking 5th in the incidence of malignancy and 4th in mortality.^[3]^ It has become a serious public health problem.^[1]^ The consumption of medical resources is increasing at an alarming rate year by year in China.^[4]^ We try to take colorectal cancer as an example to conduct an in-depth study on the cost of tumor treatment, so as to provide scientific basis for medical insurance reform.

With the constant growth of residents’ health needs, the implementation of the medical insurance system and the rise of drug prices, hospitalization costs have increased far faster than the development rate of the national economy in China. The research of hospitalization costs is one of the key points of social medical and sanitary resources.

Health insurance play an important role in medicine service in every country and the type of health insurance may be important to the cancer patient survival. Because health insurance influences the quality of health care.^[5]^ Medical security system was established in the 1950s in China. There are 2 important public health insurances, the Urban Employee Basic Medical Insurance (UEBMI) and the Urban Resident Basic Medical Insurance (URBMI), covered more than 80% people in China. The UEBMI covers urban employees and the URBMI covers people who are not employed, such as children, students, and unemployed residents.^[6]^ The “Zhanjiang model” of medical insurance is a successful example in China. The “Zhanjiang model” of medical insurance has been praised by Chinese Premier Li Keqiang on various occasions.

Research on the behavior of patients with different types of medical insurance has already been studied. For example, Gravelle et al^[7]^ and Felder et al^[8]^ showed the relationship between the optimal quality of medical insurance, the time of insurance reimbursement, the risk sharing of economic interests, and the moral hazard afterwards. Our study provides a descriptive summary of hospitalization costs of inpatients with colorectal cancer, based on an analysis for retrospective data from a 3-tier hospital in Guangdong Province in China between 2012 and 2016.

## 2 Materials and methods

This retrospective analysis identified inpatients with at colorectal cancer between 2012 and 2016. We used the database from affiliated hospital of Guangdong Medical University, a third-grade class-A hospital in Guangdong Province in China. The hospital medical service covers about 30 million people from Guangdong Province, Guangxi Province, and Hainan Province. This database could provide a sufficient sample size for this analysis.

Patient data collected included demographics, diagnosis, International Classification of Diseases (ICD), insurance type, length of hospital stay, and hospitalization costs.

We used the SPSS (version 20.0) for statistical analysis and the significance level was set at α = 0.05. The baseline characteristics of inpatients with colorectal cancer were analyzed by descriptive statistical methods. Wilcoxon rank–sum tests (for 2 groups) and the Kruskal–Wallis test (for more than 2 groups) were used to account for non-normally distributed data.^[9]^

## 3 Results

### 3.1 Demographics of inpatients

Overall, there were 8021 patients with colorectal cancer requiring hospitalization during 2012 to 2016. Of the total, the patients’ mean age was 60.04 ± 13.37. The males and females comprised 59.46% (4769 patients; mean age: 61.80 ± 13.28) and 40.54% (3252 patients; mean age: 57.45 ± 13.08), respectively. There are approximately 30.81% were covered by the UEBMI and about 58.00% were covered by the URBMI, respectively. The rest, about 11.20%, paid all by themselves without insurance. The numbers of inpatient have shown a rising trend over the 5 years (increase from 1402 to 1865). (Table 1)

**Table 1:**
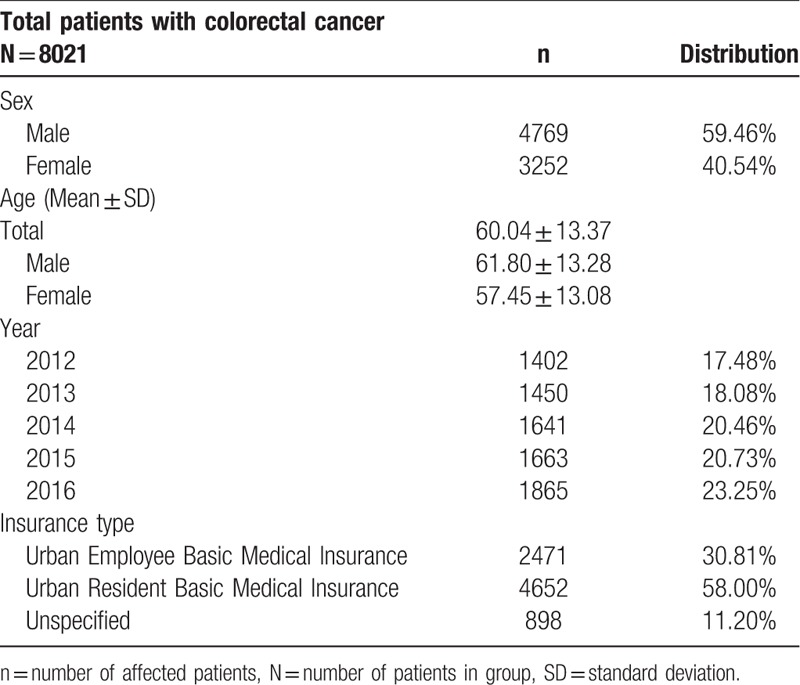
Demographics of patients with colorectal cancer.

### 3.2 Length of hospital stay and hospitalization costs associated with insurance type

The overall mean length of hospital stay was 11.35 days. The mean length of hospital stay was not similar across sexes (*P* = .039) and statistically significant difference was found for insurance status (*P* < .001). The mean length of hospital stay among the UEBMI was 11.96 days, while the mean length of hospital stay for the URBMI was 10.87 days.

For per-admission costs, the mean hospitalization costs for inpatient with colorectal cancer was ¥16,279.58. For sexes, the mean hospitalization costs were higher for men than for women (¥16,570.04 versus ¥15,853.62, respectively), and they were statistically significantly different (*P* = .049). Mean hospitalization costs were different among the UEBMI, the URBMI and Unspecified (¥17,114.58, ¥15,555.05, and ¥17,735.30, respectively; *P* < .001). (Table 2)

**Table 2:**
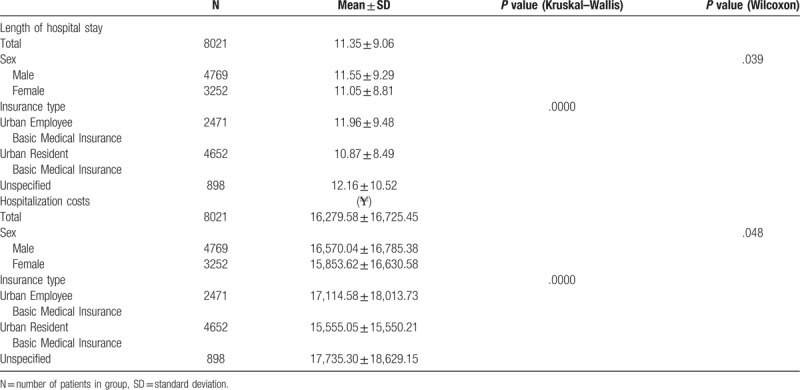
Length of hospital stay and hospitalization costs by patient characteristics and insurance types, 2012 to 2016.

### 3.3 Mean length of hospital stay and mean hospitalization costs

**Figure 1:**
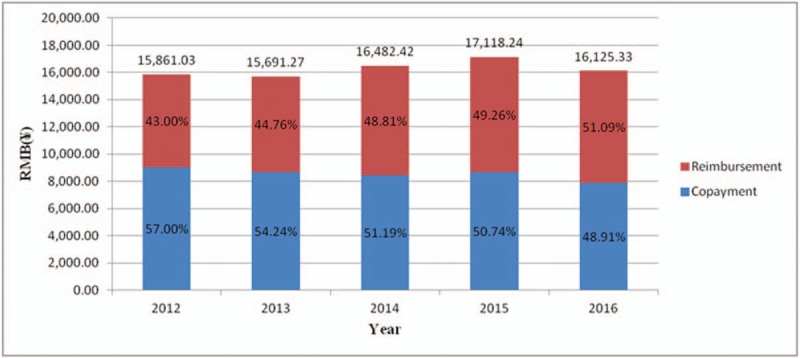
Mean hospitalization costs and copayment of patients with colorectal cancer from 2012 to 2016.

It showed that the number of inpatients was increased, but mean length of hospital stay was decreased. From 2012 to 2016, the mean length of hospital stay showed a decrease of approximately 12.5%, from 12.22 days in 2012 to 10.69 days in 2016. In contrast, per-day costs showed a trend of increase between 2012 and 2016 (16.1% increase from ¥1190.94 to ¥1382.50). In the meantime, the estimated mean hospitalization costs showed an increase of almost 7.9% from 2012 to 2015. But a decrease occurred between 2015 and 2016 (Fig. 1). As the hospitalization costs is increased, insurance reimbursement increased, while the patient proportion of the total cost decreased. (Table 3)

**Table 3:**
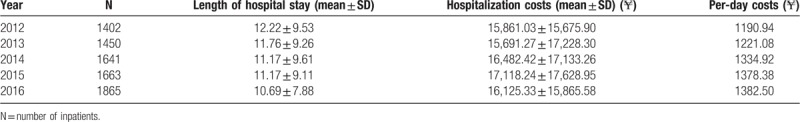
Mean length of hospital stay and mean hospitalization costs of colorectal cancer in 2012 to 2016.

### 3.4 Yearly trends in hospitalization costs by different insurance types

In 2012, hospitalization costs by UEBMI and URBMI for colorectal cancer were approximately ¥15,634.24 and ¥15,424.00, respectively. By 2016, hospitalization costs by the UEBMI and URBMI for colorectal cancer were approximately ¥17,064.13 and ¥15,383.04, respectively. It showed that hospitalization costs by the UEBMI was on the up trend, while hospitalization costs by URBMI was on the stable trend and hospitalization costs by Unspecified was on a trend of fluctuations in different years. (Table 4)

**Table 4:**
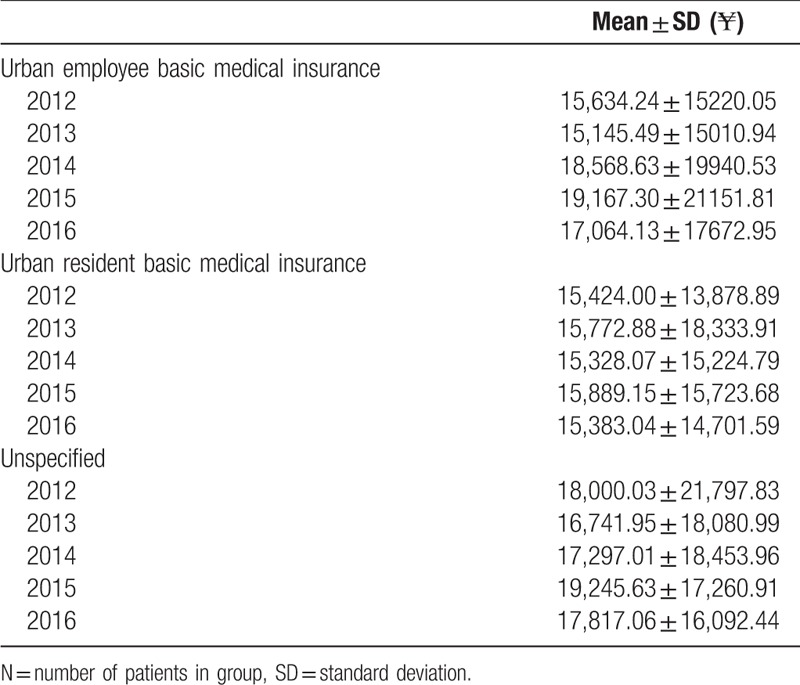
Yearly trends in hospitalization costs by different insurance types.

### 3.5 Constituent ratio of hospitalization costs

Medical costs were divided into 4 parts, drug costs, test costs, treatment costs, and others. Over the 5 years, the drug costs showed a slight decrease of approximately 6.91%, from 41.67% in 2012 to 34.76% in 2016. In contrast, treatment costs showed a trend of increase between 2012 and 2016 (9.82% increase from 14.42% to 24.24%). In the other hand, test costs revealed a trend of fluctuations. (Table 5)

**Table 5:**
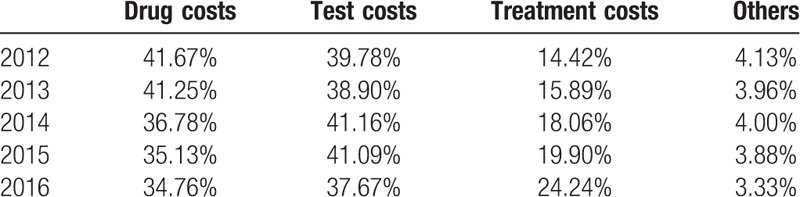
Constituent ratio of hospitalization costs.

## 4 Discussion

This retrospective database study analyzed data from 2012–2016 to estimate hospitalization costs and length of hospital stay due to colorectal cancer. The mean length of hospital stay was 11.35 days. Over the 5 years, the mean length of hospital stay revealed a slight decrease from 12.22 days in 2012 to 10.69 days in 2016, while per-day costs showed a trend of increase between 2012 and 2015 (increase from ¥1190.94 to ¥1382.50). The mean length of hospital stay was statistically significant difference was found for sexes (*P* = .039) and insurance status (*P* < .001). The mean hospitalization costs were ¥16,279.58. Mean hospitalization costs were different among the UEBMI, the URBMI and Unspecified (*P* < .001).

The hospitalization costs of tumor disease can easily lead to family disaster, which brings great financial burden to family and society. Studies have also shown that cancer is not only a serious health hazard, but also a costly one.^[10,11]^ The high cost of treating cancer patients is an international problem.^[12]^

A few previous studies have been reported to estimate on hospitalization costs and length of hospital stay for patients with colorectal cancer in China. Retrospective data on hospitalization costs and length of hospital stay from 2006 to 2011 for 1381 patients who were hospitalized for colorectal cancer in Shanxi Province, China, were analyzed by Yang et al.^[13]^ The length of hospital stay for colorectal cancer was 19 days (median). The hospitalization cost was ¥19,267.92 (median). It also showed an increasing trend of hospitalization costs was noticed year by year. Moreover, the higher drug costs were, the more hospitalization costs were spent. This may associate with drug-maintaining-medicine. It revealed that hospitals may make up for the lack of government input through using drug. Similarly, Jia^[14]^ analyzed data from 3046 patients with colorectal cancer from Beijing in 2008. The study concluded that hospitalization costs can be reduced by shortening the length of hospital stay and promoting quality of diagnosis and treatment.

In different insurance types, the mean length of hospital stay was not similar across sexes (*P* = .039) and statistically significant difference was found for insurance status (*P* < .001). The mean hospitalization costs by the UEBMI was higher than that by the URBMI. Moreover, the proportion of insurance reimbursement by the UEBMI is more than that by the URBMI. Medical insurance helps people to overcome barriers of treatment, but different medical insurances potentially bring overtreatment and resources waste in a way.

In constituent ratio of hospitalization costs, although drug costs and test costs have a downward trend, they still account for a high proportion. Before the 19th National Congress of the Communist Party of China, the most hospitals’ funding was profited from overpriced drugs. It resulted in heavy burden on the patients. But with abolishing markups on pharmaceuticals, the proportion of drug costs decreases gradually. The test costs also had a high proportion, because of use equipment, such as nuclear magnetic resonance (NMR) and computed tomography (CT) which is quiet important for doctors to evaluate disease and prognosis. Moreover, the treatment costs increased year by year. It revealed that values of certain medical services, such as skills and labor of medical staff, increased gradually.

Therefore, improving the quality of diagnosis and treatment, reducing the medical expenses by controlling the drug costs, and shortening the length of hospital stay can reduce the heavy financial burden of patients with colorectal cancer. On the other hand, clinical pathway management is an effective and effective way to manage the medical expenses of hospitalized patients.^[15]^ Using simple and effective screening technology is also beneficial to reduce medical expenses.^[16]^ Overall, despite the costs, all the above should hopefully see a fall in mortality of colorectal cancer.

An important advantage of our study is that it used recent hospital data to estimate on hospitalization costs and length of hospital stay due to colorectal cancer. The study can provide details that can be used in economic evaluation. It also has some limitations included single hospital and small sample. Also, subgroups were just analyzed by sex and insurance type. But it was not analyzed subgroups based on age or stage. In addition, inflation is not considered.

## 5 Conclusion

Our study used recent and cross-sectional data to provide more accurate estimates on the hospitalization costs of colorectal cancer in China. The study showed that hospitalization costs of the UEBMI was higher than the URBMI, and the length of hospital day of the UEBMI was longer than the URBMI. The proportion of the hospitalization costs reimbursed by health insurances increased. For inpatients with UEBMI, it possibly lead to over treatment and the medical expense rise which result in medical resources waste and significant society costs. In the case of China, the hospitalization costs remain unaffordable for most inpatients with colorectal cancer, especially for inpatients without health insurance. It also showed that drug costs and test costs play important roles in hospitalization costs. Reducing the cost of drugs and test can make more patients receive better treatment. This study fails to clarify how to maximize benefit of inpatients with colorectal cancer. Therefore, we still have to make further study.

## Acknowledgments

The authors would like to thank the Guangdong Medical University, China and Affiliated Hospital of Guangdong Medical University, China for providing database and other research support. We also would like to thank for setting up and managing the database dedicated to the collection of such a large amount of data.

## Author contributions

**Conceptualization:** Zhong-Fang Zhang.

**Data curation:** Li-Zhong Liang.

**Formal analysis:** Zhen-Yi Huang.

**Funding acquisition:** Li-Zhong Liang, Zhong-Fang Zhang, Qilian Liang.

**Investigation:** Hui-Jie Zhang, Shao-Ang Cheng, Xiao-Xia Peng.

**Methodology:** Zhen-Yi Huang.

**Project administration:** Qilian Liang.

**Resources:** Li-zhong Liang, Hui-Jie Zhang, Xiao-Xia Peng.

**Software:** Hui-Jie Zhang, Shao-Ang Cheng, Xiao-Xia Peng.

**Supervision:** Zhong-Fang Zhang, Shao-Ang Cheng.

**Validation:** Zhong-Fang Zhang.

**Visualization:** Hui-Jie Zhang, Shao-ang Cheng, Xiao-xia Peng.

**Writing – original draft:** Gao-Le Yuan.

**Writing – review & editing:** Zhong-Fang Zhang, Qilian Liang.

Qilian Liang orcid: 0000-0003-3245-1239.
